# Comparative genomic analysis of the *PAL* genes in five Rosaceae species and functional identification of Chinese white pear

**DOI:** 10.7717/peerj.8064

**Published:** 2019-12-02

**Authors:** Guohui Li, Han Wang, Xi Cheng, Xueqiang Su, Yu Zhao, Taoshan Jiang, Qin Jin, Yi Lin, Yongping Cai

**Affiliations:** School of Life Science, Anhui Agricultural University, Hefei, China

**Keywords:** Phenylalanine ammonia lyase (PAL), Rosaceae plants, Expression analysis, Lignin synthesis, Pear

## Abstract

Phenylalanine ammonia lyase (PAL) plays an important role in the biosynthesis of secondary metabolites regulating plant growth response. To date, the evolutionary history of the *PAL* family in Rosaceae plants remains unclear. In this study, we identified 16 *PAL* homologous genes in five Rosaceae plants (*Pyrus bretschneideri*, *Fragaria vesca*, *Prunus mume*, *Prunus persica*, and *Malus* × *domestica*). We classified these *PAL*s into three categories based on phylogenetic analysis, and all *PAL*s were distributed on 13 chromosomes. We tracked gene duplication events and performed sliding window analysis. These results revealed the evolution of *PAL*s in five Rosaceae plants. We predicted the promoter of the *PbPAL*s by PLANT CARE online software, and found that the promoter region of both *PbPAL1* and *PbPAL3* have at least one AC element. The results of qRT-PCR analysis found that *PbPAL1* and *PbPAL2* were highly expressed in the stems and roots, while expression level of *PbPAL3* was relatively low in different tissues. The expression of *PbPAL1* and *PbPAL2* increased firstly and then decreased at different developmental periods of pear fruit. Among them, the expression of *PbPAL1* reached the highest level 55 days after flowering. Three *PbPAL*s were induced by abiotic stress to varying degrees. We transfected *PbPAL1* and *PbPAL2* into *Arabidopsis thaliana*, which resulted in an increase in lignin content and thickening of the cell walls of intervascular fibres and xylem cells. In summary, this research laid a foundation for better understanding the molecular evolution of *PAL*s in five Rosaceae plants. Furthermore, the present study revealed the role of *PbPAL*s in lignin synthesis, and provided basic data for regulating lignin synthesis and stone cells development in pear plants.

## Introduction

Pear, a major fruit variety of the Rosaceae, one of the most important deciduous fruit trees in the world. ‘Dangshan Su’ pear (*Pyrus bretschneideri* cv. Dangshan Su), originating in Dangshan County, Anhui Province, China, which is the most widely cultivated pear variety at present in China (Konarska, 2013). But there is a defect in the variety: the content of stone cell mass in its fruit is high and its diameter is large, which restricts the development of ‘Dangshan Su’ pear industry.

The content and size of the stone cell mass is one of the key factors affecting pear fruit quality. The content and diameter of the stone cell group significantly affects the meat quality, and the size of the stone cell group is highly negatively correlated with the fruit’s qualit (Jin et al., 2013b; Cheng et al., 2018). In the development of ‘Dangshan Su’ pear fruit, the two peak of lignin content appeared before the peak of the stone cell content and the maximum diameter of the stone cell mass (Cheng et al., 2017). A large amount of lignin synthesis may be material preparation for the development of stone cells. The development of stone cells is closely related to lignin biosynthesis, deposition, and polymerization (Wu et al., 2013; Yan et al., 2014). Therefore, studies of the structural and regulatory genes involved in phenylpropane metabolism pathway have been helpful in understanding the synthesis and regulatory mechanisms related to plant secondary metabolites. These studies have also provided a basis for further genetic engineering to modify plant cell metabolic flow and to improves crops.

Phenylalanine ammonia lyase (PAL) plays a significant role in phenylpropanoid metabolism pathway. PAL, as the first key enzyme in phenylpropanoid biosynthesis, catalyzes the conversion of L-phenylalanine to cinnamic acid, linking primary metabolism with secondary metabolism, which is a speed-limiting step in phenylpropanoid metabolism (Wang et al., 2014b). PAL is widely found in various plants. Since the discovery of the first *PAL* in barley, more and more *PAL*s have been cloned from many higher plants, such as *Rhus chinensis* (Ma et al., 2013), *Dendrobium* (Jin et al., 2013a), *Lycoris radiata* (Jiang et al., 2013). Interestingly, *PAL*s also have been successfully cloned, expressed in some liverworts (Yu et al., 2014) and fungi (Yun et al., 2015). PAL is the fulcrum enzyme controlling primary metabolism to secondary metabolism in the phenylpropanoid metabolic pathway. This metabolic pathway not only produces well-studied flavonoids, concentrated tannins and lignin, but also produces less-studied benzene compounds and phenolic glycosides.

PAL is encoded by a polygenic family and has different numbers of members in different plants, for example, *Brachypodium distachyon* (eight PALs), *Populus trichocarpa* (5 PALs), and *Eucalyptus grandis* (nine PALs) (Jaime et al., 2016; Shi et al., 2010; Chong et al., 2018). In a previous study, the importance of *PAL*s in plant development and defense has been confirmed. Recently, four *PAL*s were identified, expressed and characterized in *Arabidopsis thaliana.* Among them, *AtPAL1* and *AtPAL2* are mainly expressed in most tissues, while the expression level of *AtPAL3* and *4* are relatively low in different tissues (Cochrane, Davin & Lewis, 2014). Previous studies have shown that the role of AtPAL protein in PAL double mutants is redundant, and the lignin content of *A. thaliana* plants with *pal1 pal2* double mutant decreased significantly, tannic acid in seed coat was lack of concentration (Chong et al., 2018). *AtPAL1* and *AtPAL2* sensitive strongly to abiotic environmental factors, such as, temperature, UV-B, and play a redundant role in the synthesis of flavonoids and lignin (Huang et al., 2010). In contrast, the expression level of *PAL*s showed significant difference in poplar. For example, *PtPAL1* and *3* are expressed in most tissues, which they are mainly responsible for the production of concentrated tannins, flavonoids and other phenolic metabolites, whereas *PtPAL2*, *4* and *5* were found to be mainly expressed in xylem tissues. It is speculated that they may be mainly responsible for lignin synthesis in poplar trees (Kao, Harding & Tsai, 2002; Shi et al., 2013). Therefore, it can be seen that *PAL* is indispensable in the lignin synthesis.

At present, the *PAL* family is screened and identified in *A. thaliana*, *Camellia sinensis* and other plants, and their critical roles in the formation of catechins, flavonols and their derivatives have also been clarified (Wu et al., 2017). However, genome-wide analysis of the *PAL* family in Rosaceae plants is rarely reported. The function of *PAL* family in lignin synthesis is also rarely studied, and there is no report in the study of the pear. We know nothing about which members of the pear *PAL* family are involved in lignin synthesis. To fill this gap, we screened three *PAL* members from pear genome and analyzed them systematically. It includes amino acid property, gene structure, conservative motif, phylogenetic relationship, *cis*-acting elements. Combined with lignin content determination and spatiotemporal expression pattern analysis, the candidate *PAL* members associated with lignin synthesis were identified in order to lay a foundation for the mechanism of lignin synthesis and control the development of stone cells.

## Materials and Methods

### Plant materials and treatments

The buds, stems, leaves, flowers, roots and fruits were collected from 60 years old pear trees, which managed on a farm in Dangshan, Anhui, China. Uniformly sized fruits were collected at eight time points: 15 DAF (days after flowering), 39 DAF, 47 DAF, 55 DAF 63 DAF, 79 DAF, 102 DAF and 145 DAF. All of the fruit samples were stored at −78 °C for further use.

To investigate the effect of hormone treatments on the gene expression levels of genes related to lignin biosynthesis pathway in pear fruit, we seected pests-free of the pear trees of same age and plant height. The concentration of the hormone treatment (the 0.5 mmol/L abscisic acid (ABA), 0.5 mmol/L methyl jasmonate (MeJA), or 0.2 mmol/L salicylic acid (SA)) were sprayed onto the fruits 39 DAF (Cheng et al., 2019). All samples were treated for 3 h under the same conditions. The pear flesh was weighed about 100 g and frozen at −20 °C for 24 h. The lignin content was measured using the Klason method (Cai et al., 2010).

### Acquisition and identification of *PAL* family members

In this study, we have identified the number of *PAL* members in five Rosaceae plants. Pear genome database was obtained from (http://gigadb.org/dataset/100083) (Wu et al., 2013). The sequence information of *Prunus mummer* (mei), *Malus domestica* (apple), *Prunus persica* (peach) and *Fragaria vesca* (strawberry) gene were obtained from the Phytozomes database (https://phytozome.jgi.doe gov/pz/portal.html) (Jung et al., 2014). Initially, we acquired the Hidden Markov Model (HMM) profile of PAL proteins from the Pfam database (http://pfam.sanger.ac.uk/). Subsequently, we utilized the HMM profile as a query to identify all PAL-containing sequences by searching against the three of Rosaceae species genomes (E-value = 0.001). Then, all candidate *PAL*s are validated using Pfam (http://pfam.xfam.org/) (Punta, 2015) and SMART database (http://smart.embl-heidelberg.de/) (Letunic, Doerks & Bork, 2012) to confirm that they contain core domains. Finally, we removed all potentially redundant PAL sequences according to the results of the sequence alignments.

### Conserved motif, *cis*-element and feature analyses of the *PAL*s

The online analysis tool ExPASy (http://web.expasy.org/compute_pi/) is used to predict the isoelectric point (pI) and protein molecular weight of (kDa) of each PAL based on their amino acid sequences. Prediction of subcellular localization was performed using the online tool MBC (http://cello.life.nctu.edu.tw/). Phylogenetic trees were constructed by the N-J method (bootstrap = 1,000) in MEGA5.0 software (Tamura et al., 2011). Analysis of exons and introns were carried out using the gene structure display server (GSDS) program (Liu et al., 2016). Conserved protein motifs were confirmed by MEME (http://meme-suite.org/) (Bailey et al., 2015), which following parameters: the maximum number of motifs is 20, and the base length is between 6 and 200.

The 2,000 bp promoter sequence of the *PbPAL* family members were obtained from the genome database of ‘Dangshan Su’ and then the online software PLANT CARE database was employed to analyze the *cis*-acting elements in the promoter regions (Lescot et al., 2002).

### Chromosomal locations and Ka (nonsynonymous)/Ks (synonymous) analysis

The chromosomal locations of the *PAL*s in five Rosaceae plants were obtained from genome annotation documents. The data were then plotted using the Circos software (Krzywinski et al., 2009). The duplication events were categorized into segmental and tandem duplication (Cao et al., 2018). Ka and Ks were calculated by DnaSPv5.0 software (Wang et al., 2010). Sliding window analysis was also carried out using this software.

### RNA extraction and qRT-PCR analysis for *PbPAL*s

Extraction of total RNA from the fresh pear fruit was performed using a Plant RNA Isolation Kit (Tiangen, China) for qRT-PCR analysis, and the fresh pear fruit here is not from the material used for lignin determination. Next, DNA was reverse transcribed from 1 µg of RNA using the transcriptase M-MLV system (Tiangen, Beijing, China) according to the manufacturer’s instructions. Primers (Table S1) were designed for real-time quantitative PCR (qRT-PCR) using the Beacon Designer 7 software. Tubulin (GenBank accession no. AB239680.1) (Wu et al., 2013) was used internal reference. The transcript levels were measured using a CFX96 Touch™ Real-Time PCR Detection System (BIO-RAD). The total volume of the reaction mixture was 20 µL, consisting of 10 µL of SYBR Premix Ex Taq II (2*x*), 2 µL of template cDNA, 0.8 µL of the forward and reverse primers and ddH_2_O up to 20 µL. The relative expression levels of the genes were calculated using the 2^−ΔΔ*CT*^ method.

### Arabidopsis transformation

The full-length CDS of *PbPAL1* (GenBank: MF346686) and *PbPAL2* (GenBank: MF346687) were cloned from pear (Table S2). The correct pMD18-T-*PbPAL* plasmid and pCAMBIA1304 (GenBank: AF234300.1) vector plasmid were digested by restriction endonuclease *Bgl* II and *Spe* I (Takara, Japan) (Table S3), respectively. Subsequently, the recombinant eukaryotic expression plasmid pCAMBIA1304-*PbCPAL* was constructed and successfully obtained by ligation with T4 DNA ligase. Transformation of recombinant plasmid pCAMBIA1304-*PbPAL* into *Agrobacterium* tumefaciens EHA105. The *A. tumefacien* culture at 28 °C in medium with recombinant plasmid pCAMBIA1304-*PbPAL*. The bacteria were suspended in infection buffer (0.02% Silwet L-77, 1/2 MS, 5% sucrose). The OD_600_ value of the infection solution was approximately 0.7–0.8, which is acceptable for subsequent infection.

The seeds of *A. thaliana* were sterilized (75% ethanol for 1 min, 10% sodium hypochlorite for 13 min). After 4 washes with sterile water, the seeds were evenly sown on MS solid medium plates containing hygromycin. After approximately 15 days, seedlings with 4 true leaves were transplanted into nutrient soil for further cultivation.

Selected pCAMBIA1304*-PbPAL* plants and wild type plants of some lotus leaves were grown for approximately 20 days. At the same time, leaf of DNA was extracted and tested by PCR with *gfp* specific primers (Table S4).

### Determination of lignin content in *A. thaliana*

Dried mature stems were collected after removal of the leaves. The lignin levels of wild-type and overexpression plants were determined using the acetyl bromide method (Anderson et al., 2015). The lignin content was expressed as a percentage (calculated lignin content/calculated dry weight of test sample × 100%) (Tian et al., 2017).

### Histochemical staining of *A. thaliana* inflorescence stems

Inflorescence stem segments of 60-day-old transgenic T_3_ generation and wild type *A. thaliana* plants in the same position were collected (the sections were from the bottom approximately 4 cm of the inflorescence stems). The samples were fixed in 50% (v/v) ethanol, 100% (v/v) glacial acetic acid and 95% (v/v) methanol solution overnight and then embedded in paraffin for lateral and transverse sectioning at a thickness of 4 µm using a pathology slicer (RM2016). Plant tissue sections are placed in the dye solution (1% toluidine blue and Wiesner reagent) for about 2–5 min, washed with water, and the slices are placed in the oven and baked. Transparent sealing with neutral gum, and then directly observed with a microscope (Pradhan & Loque, 2014). Photographs were taken under a binocular microscope.

## Results

### Collection and identification of *PAL*s in five Rosaceae plants

Based on the HMM sequence on the Pfam website (http://pfamxfamorg/) and BLASTP strategies, *PAL* family members were identified from five Rosaceae species. The target sequence was compared with the DNAMAN software in the genome database, then remove repetitive redundant sequences. Finally, in our study, we identified 16 non-redundant and complete *PAL*s in five Rosaceae species (Table 1). The correspondent proteins displayed that their lengths, molecular weights, isoelectric points (pI), were within the ranges of 414–753 amino acids, 44.42–87.75 kDa, 5.79–8.79, respectively (Table 1).

**Table 1:** Sequence information of the *PAL* family members of five Rosaceae plants.

**Species**	**Gene name**	**Gene ID**	**Length (aa)**	**Mw (kDa)**	**pI**	**Chromosome**	**Strand**
	MdPAL1	MDP0000668828	720	78.55	6.09	Chr1	cyto
	MdPAL2	MDP0000787168	643	69.90	6.39	Chr8	cyto
**Apple**	MdPAL3	MDP0000261492	720	78.15	6.29	Chr4	cyto
	MdPAL4	MDP0000388769	753	87.75	6.21	Chr12	cyto
	MdPAL5	MDP0000139075	589	63.41	6.31	Chr12	cyto
	MdPAL6	MDP0000191304	702	76.16	6.18	Chr4	cyto
	PmPAL1	Pm030127	717	77.92	6.10	Chr8	cyto
**Mei**	PmPAL2	Pm018524	719	78.18	6.19	Chr5	cyto
	FvPAL1	Fv23261	718	77.98	6.00	Chr7	cyto
**Strawberry**	FvPAL2	Fv09753	724	78.98	6.10	Chr6	cyto
	PpPAL1	Ppa002328m	686	74.63	6.28	Chr2	cyto
**Peach**	PpPAL2	Ppa002099m	716	78.00	6.10	Chr6	cyto
	PpPAL3	Ppa002878m	625	67.87	6.39	Chr2	oute
	PbPAL1	Pbr008363	720	78.15	6.29	Chr12	cyto
**Pear**	PbPAL2	Pbr008387	715	77.83	5.79	Chr3	cyto
	PbPAL3	Pbr016460	414	44.42	8.79	Chr5	cyto

### Conserved motifs and gene structure of *PAL* family members of five Rosaceae species

**Figure 1:**
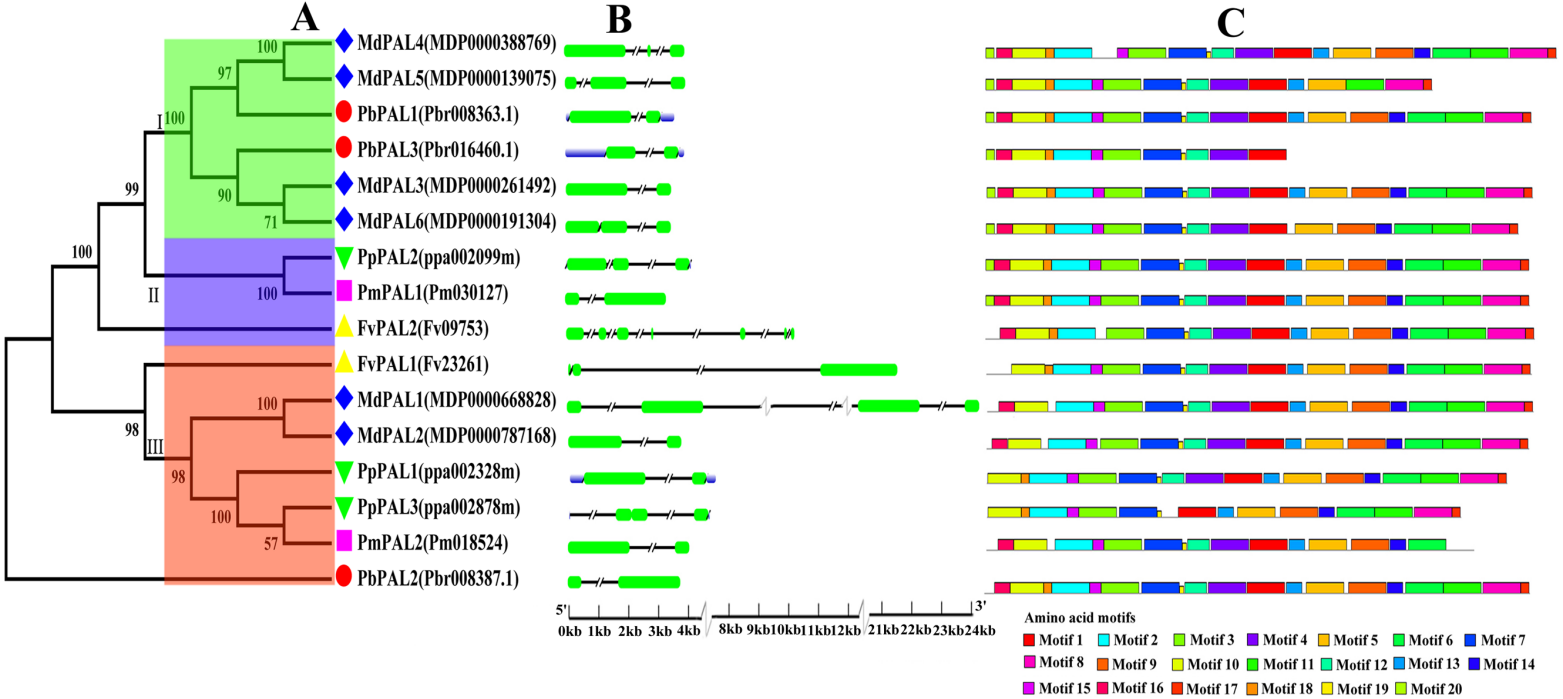
Phylogenetic relationships, and gene structures and domain of *PALs*. (A) Phylogenetic tree of *PAL* genes was conserved using MEGA5.0 by the N-J method. (B) Exon-intron organization of *PAL*s using the GSDS program. The exons and introns are indicated by arrows and thin lines, respectively. (C) Conserved domains in PAL proteins using the MEME program.

To investigate the evolutionary relationships of the *PAL* family of five Rosaceae species, we constructed a phylogenetic tree using the MEGA5.0 (Fig. 1A). Phylogenetic analysis of *PbPAL*s revealed that the existence of highly differentiated *PAL*s in *P. bretschneideri* and some other Rosaceae plants, which the 16 *PAL*s were clustered into three major clades. Conservative gene structures may provide a record of key events in the evolution of genes. Furthermore, *PAL*s structure analysis also supported clustering of occurrence groups. We found that in the same subfamily, the structure of *PAL* is usually very similar (Fig. 1B). But sometimes there are special phenomena, for example, in Cluster II members, the results shown that *FvPAL2* structure is longer and contains more than one exon and intron, while *FvPAL1* only contains three exons. Besides, the number, length and location of exons and introns are also different in *PAL*. In this study, we found that most members of *PAL*s in five Rosaceae species contain two or three exons, which means that these genes are highly conserved during evolution.

To better understand the structural diversity of PALs, we captured twenty conversed motifs in PAL with the PAL protein sequences using MEME software (Fig. 1C). The conserved motif analysis of PALs proved the reliability of the phylogenetic relationship. Moreover, our results also suggested that most of PAL proteins have similar motifs in the same subfamily. Besides, the number of motifs involved in PAL protein sequence was quite uncertain. Coincidentally, motifs 1, 2, 3, 7 and 19 were found in all PAL protein sequences of five Rosaceae species. However, some of the motifs were found to be unique to a subfamily. For example, motif 20 only was found in Cluster I. PbPAL3 had fewer motifs, indicating the PAL domain may be incomplete.

### Chromosome location and gene duplication event analysis of *PAL* members family in five Rosaceae plants

**Figure 2:**
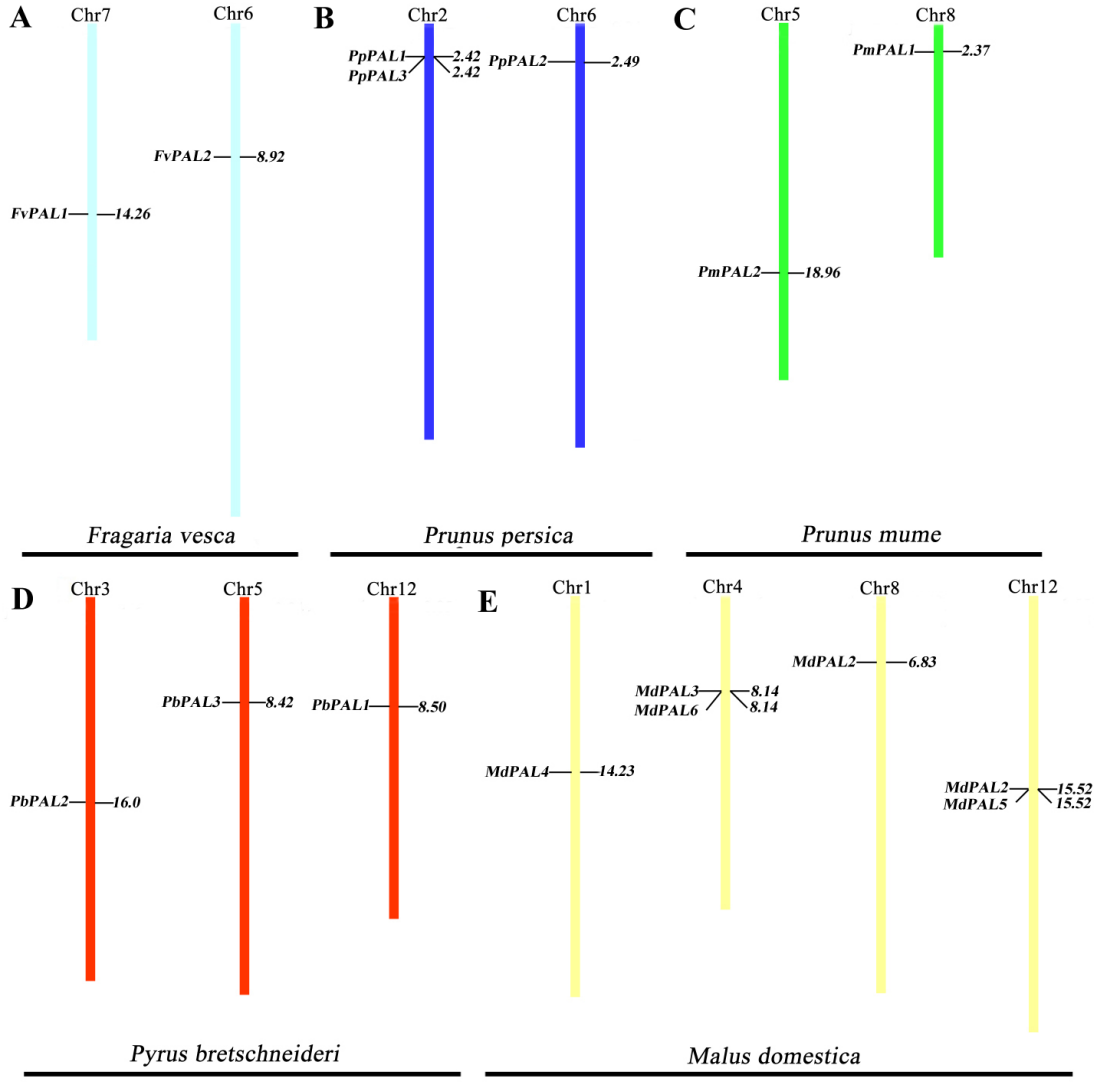
*PAL*s chromosomal location of five Rosaceae species. Chromosomal locations of *PAL* genes in *Fragaria vesca* (A), *Prunus persica* (B), *Prunus mume* (C), *Pyrus bretschneideri* (D) and *Malus domestica* (E). The chromosome number is indicated at the top of each chromosome. Different chromosome colours represent different species.

To clarify the distribution of *PAL* family members on the chromosomes of five Rosaceae species. According to the genome information of each species, and we constructed a chromosomal location map (Fig. 2). The *PAL*s are randomly distributed on 13 chromosomes. Two genes each are located on one chromosome in strawberry and plum blossom. Three genes each are located on one chromosome in *P. brestschneideri.* Two chromosomes containing three genes in *P. persica.* Four out of the 13 chromosomes harbored *MdPAL*s, with 2 (chromosomes 1and 8) possessing one *MdPAL* and 2 (chromosomes 4 and 12) possessing two *MdPAL*s.

Segmental or tandem duplication is the main way to increase the number of family members in plants. In order to further explore the driving forces of *PAL* evolution, we calculated the rate of nonsynonymous/synonymous substitution (Ka/Ks) among five gene paris. Five pairs of gene duplication events were found in sixteen *PAL*s of five Rosaceae species (Fig. S1). Generally, Ka/Ks>1 indicates positive selection and accelerates evolution; Ka/Ks<1 indicates functional constraints of negative selection. Our results showed that all Ka/Ks pairs of *PAL*s were less than 1 (Table 2), which illustrates that they have undergone strong evolutionary selection, and their functions have not been seriously differentiated. Except *MdPAL3/MdPAL6* belonged to tandem duplication, the others were segmental duplication, which indicated that the expansion of *PAL* family of five Rosaceae species was mainly due to segmental duplication events.

**Table 2:** Analysis of gene duplication events of *PAL* family members in Rosaceae species.

Paralogous pairs	Ks	Ka	Ka/Ks	Purifing selection	Duplicate type
*MdPAL1/MdPAL2*	0.2676	0.0250	0.0903	Yes	Segmental
*MdPAL4/MdPAL5*	0.1034	0.0961	0.9294	Yes	Segmental
*MdPAL3/MdPAL6*	0.1368	0.0532	0.3889	Yes	Tandem
*PbPAL2/PmPAL2*	1.9819	0.2017	0.1017	Yes	Segmental
*PmPAL1/PmPAL2*	0.0577	0.0037	0.0641	Yes	Segmental

### Promoter analysis of *PAL*s in pear

**Figure 3:**
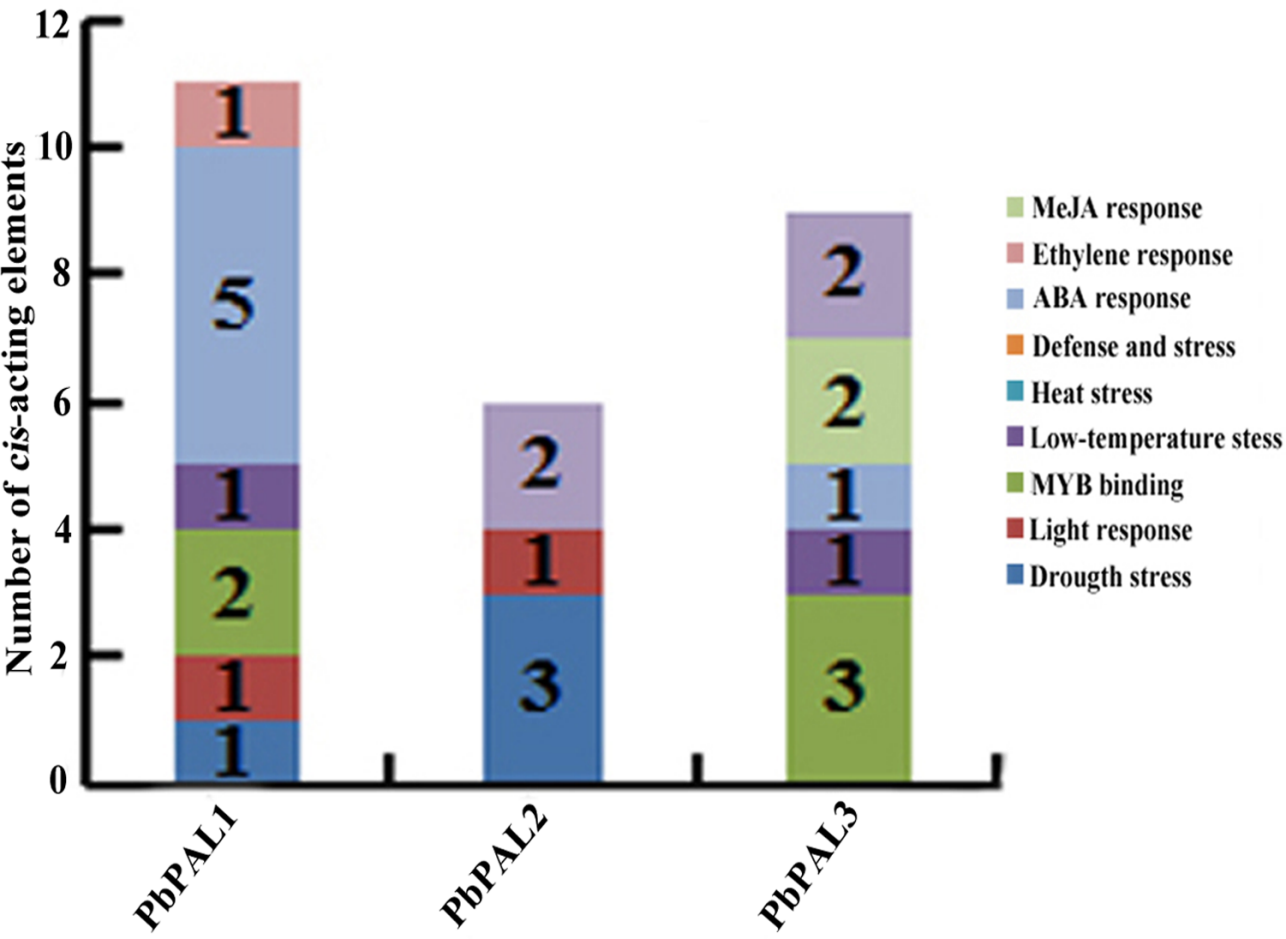
Distribution of main *cis*-elements and putative regulating factors in the promoter regions of pear *PAL* genes. Different *cis*-elements with the same or similar functions are shown in the same color.

To further understand the regulation mechanism of *PbPALs* expression, we predicted possible *cis*-acting elements using PLANT CARE online software (Table S5 and Fig. 3). It was found that the promoter of *PbPAL*s contained two types of stress response regulatory elements, such as MBS and LTR repetitive sequences, which responds to drought induction, and cold stress, respectively. Among which four kinds of hormone regulatory elements: ERE, ABRE, CGTAC-motif and TCA-element were associated with ethylene, ABA, MeJA and SA responses respectively. In addition, two members of the *PbPAL*s family contain the MRE light-responsive element, which hinted that expression of *PbPAL*s were closely related to light. Furthermore, we found that *PbPAL1* and *3* gene contains at least one AC element, AC element can activate lignin monomer synthesis gene by binding with MYB transcription factor (Patzlaff, 2010). Therefore, we proposed that expression of *PbPAL*s are closely related to lignin formation.

### Phylogenetic analysis of *PAL*s in pear and other plants

**Figure 4:**
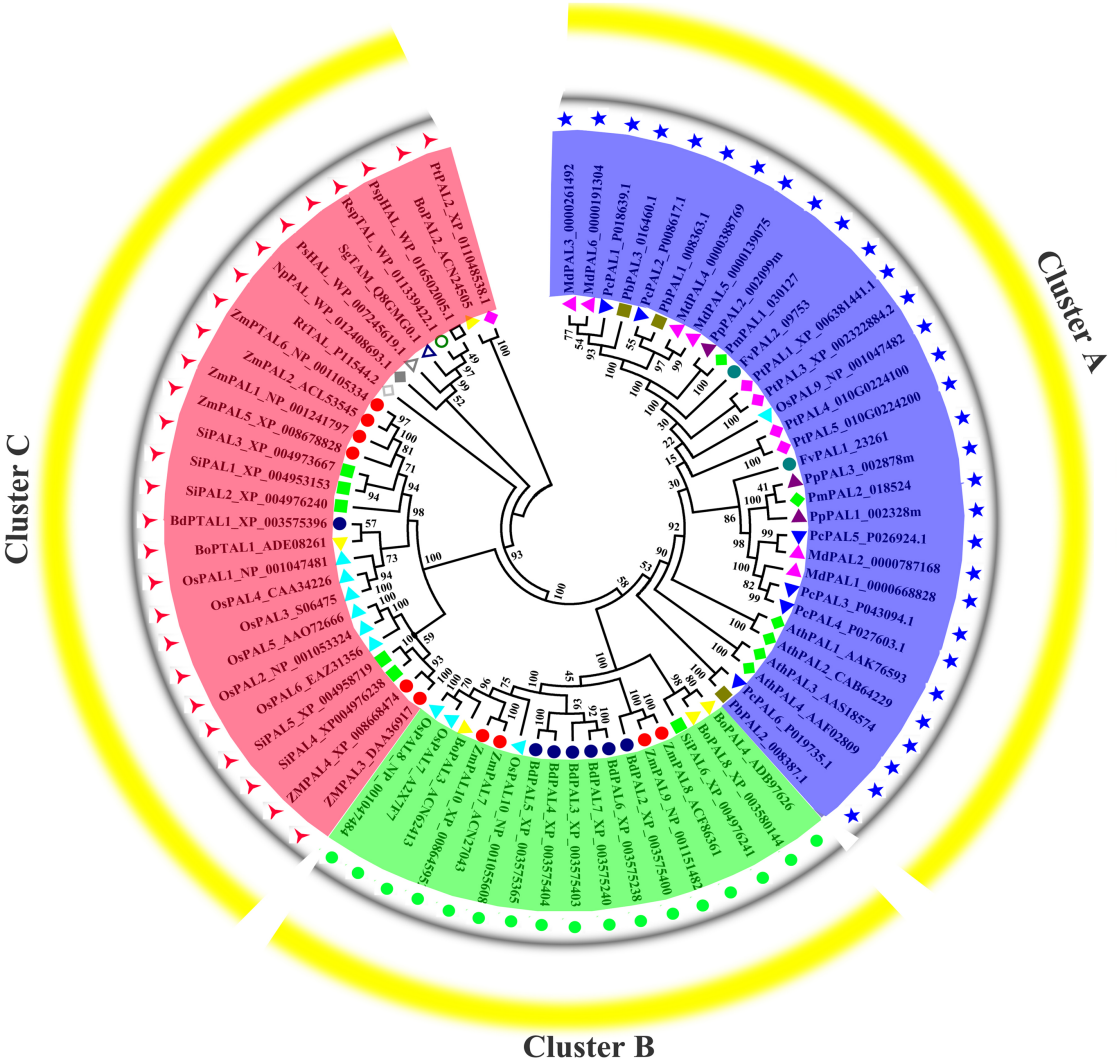
Phylogenetic analysis of *PAL*s constructed by the Neighbor-Joining method. Phylogenetic tree of PTAL and PAL in plants and fungi, and TAL, tyrosine ammonia-mutase (TAM) and histidine ammonia-lyase (HAL) in bacteria. These PAL sequences were clustered into three groups; purple, green and red lines indicate the three subfamilies of the PAL proteins.

Previous research shown that *NnPAL1* as an ancient member of the *PAL* family, and was found to be a polybasic origin in the evolution of PAL in angiosperms (Wu et al., 2014; Wu et al., 2017). To investigate the phylogenetic relationships of *PbPAL*s with other plants and bacterias, which a neighbor-joining tree was created. The phylogenetic tree clustering results showed that *PAL*s of fifteen species could be divided into three well-supported families (Fig. 4). Previous works have shown that the *PAL* family was divided into a subfamily of *A. thaliana*, which was consistent with our classification results (Barros et al., 2016). During the evolution of *PAL*, the recurrence of specific pedigrees occurred in *A. thaliana*, *P. trichocarpa* and *Selaginella moellendorffii*. This is supposed to be a universal phenomenon that promotes the diversity of polygenic families. In this study, the *PbPAL*s were intimately related to dicotyledon plant PAL and belongs to the group. However, the three *PbPAL*s were aggregated with each other and form a different subgroup. Interestingly, just as the results of *PbPAL*s classification are resemble, most of plant *PAL*s are clustered by species, and *PAL*s are in one species are closer to each other than their homologues in another. Based on this evidence, PAL diversity occurs independently in each species. In addition, we found *PtPAL2* and *BoPAL2* are closer to tyrosine ammonia-mutase (TAM) and histidine ammonia-lyase (HAL). Based on peptide sequence similarity, we speculated that *PtPAL2* and *BoPAL2* may could encode putative ammonia-lyases.

### Expression profiles of pear *PAL*s at different tissues and developmental stages of fruits

**Figure 5:**
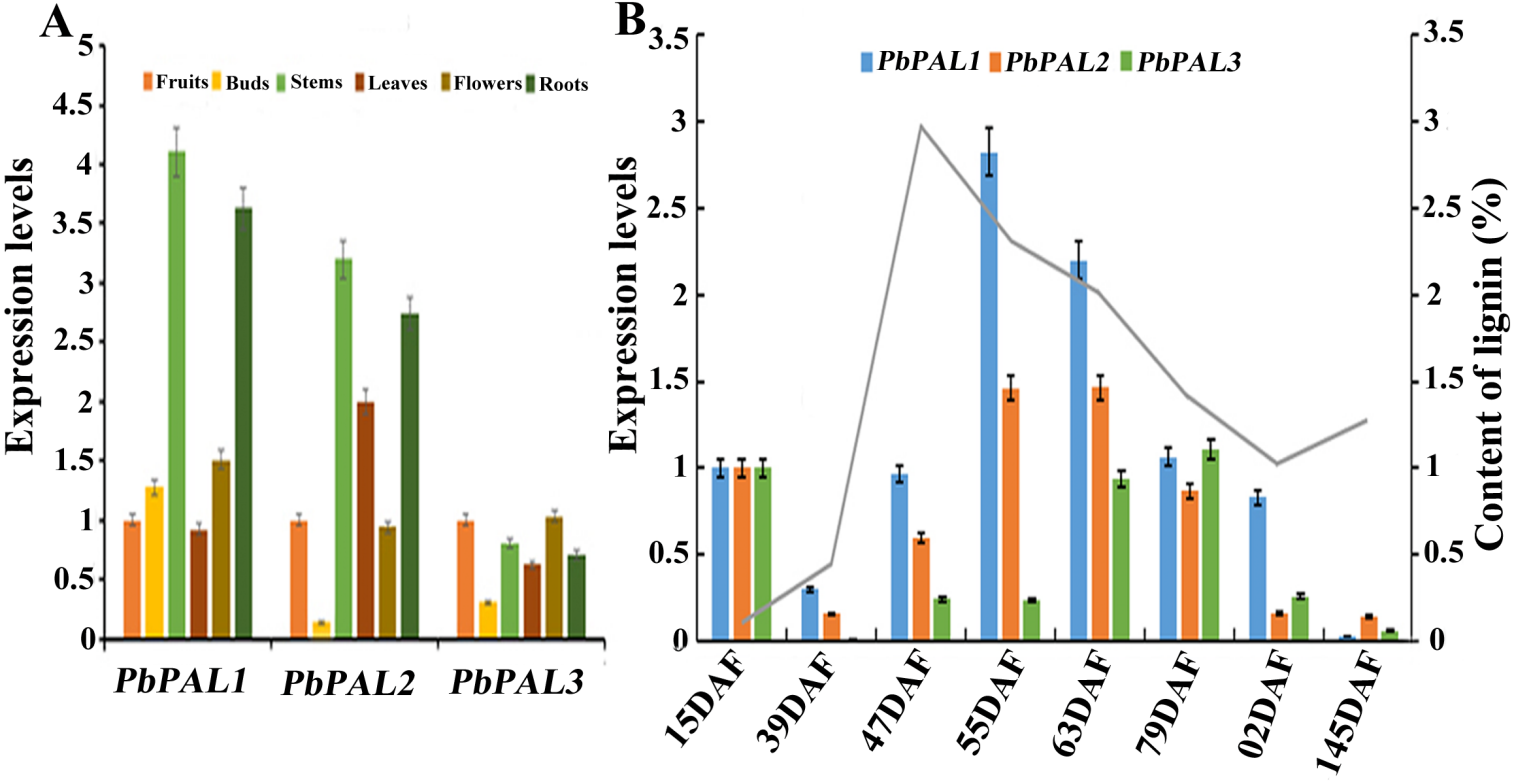
Expression analysis of *PbPAL*s at various tissues of pear (A) and at different stages of fruit development (B). Fifteen days after flowering (DAF), 39 DAF, 47 DAF, 55 DAF, 63 DAF, 79 DAF, 102 DAF and mature stage (145 DAF), respectively. *Y*-axes on the left indicates the relative gene expression levels; (*X*-axis) by bar charts, and the *Y*-axes on right showed content of lignin during fruit development with line charts. Each histogram represents the mean value and the bar ± standard error of three biological replicates. The material used to determine the level of expression is not derived from the material used to determine lignin.

The potential functions of gene families can be probed by means of gene expression analysis (Cao et al., 2016). To further describe the function of the pear *PAL*s, and comparative gene expression analysis was carried out in different tissues or organs (leaves, stems, flowers, roots and buds) (Fig. 5). The results showed that transcript levels of *PbPAL1* and *2* were higher in lignified tissues (roots and stems) than in less lignified tissues (leaves, buds and flowers) (Fig. 5A). Therefore, *PbPAL1* and *2* are highly expressed in stems and roots, and we conjectured that they may be involved in lignin biosynthesis in pear. While expression level of *PbPAL3* was relatively low in different tissues. These results suggest that different *PbPAL*s may play key roles in the development of specific tissues.

The content of stone cell is an important factor affecting the quality of pear fruit. As one of the main components of stone cell wall, lignin synthesis directly affects the formation of stone cells rich in pear fruits (Cai et al., 2010; Jin et al., 2013b). Moreover, the change of lignin content is also closely related to the change of stone cell content. Afterward, the expression profiles of these *PbPAL*s at different the stages of fruit development were also surveyed by using qRT-PCR (Fig. 5B). Previous studies showed that the content of stone cell and lignin in pear fruit first increase and then decrease during fruit development, reaching the peak at 55 DAF (Cai et al., 2010). It is noteworthy that the expression levels of *PbPAL1* and *2* were proporational to the content of stone cell and lignin in pear fruits, indicating that these genes might be related to lignin aggregation and stone cell formation in pear fruits. This study implying that *PbPAL1* and *2* are closely related to lignin synthesis and stone cell development. While *PbPAL3* was highly expressed at the 79, 102 and 145 DAF, indicating that this gene might play important roles in the mature stage of pear fruit development.

### Differential expression *PbPAL*s under hormonal treatments

**Figure 6:**
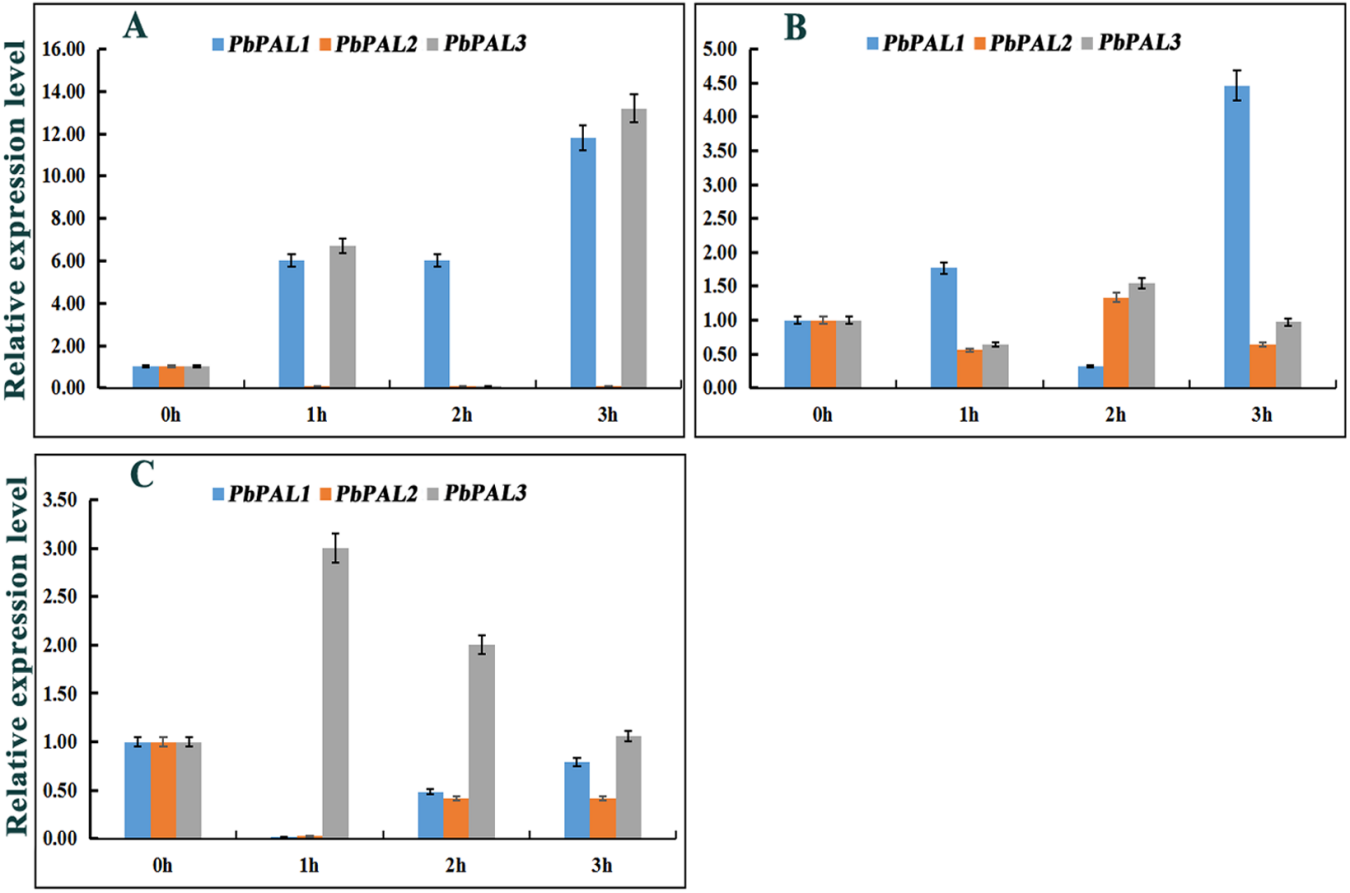
Hormone response pattern analysis of *PbPALs*. *PbPALs* expression in pear fruits in response to exogenous hormone (A, ABA; B, MeJA; C, SA) treatments for 0, 1, 2, and 3 h. The gene expression data of *PbPALs* at 0 h, 1 h, 2 h and 3 h were obtained through reverse transcription-quantitative real-time polymerase chain reaction (qRT-PCR). The expression data of each gene at 0 h was used as a control sample to show the relative expression level.Each qRT-PCR analysis was performed in triplicate. Error bars indicate the standard deviation of three replications.

Previous studies have shown that the expression of *PAL*s are subjected to abiotic stress (Scott et al., 2004). However, information on *PAL*s involvement in pear hormone response is limited. Previous studies have found that spraying exogenous hormones on the pear fruits can regulate stone cell development and lignin synthesis in pear fruits to a certain extent (Yang et al., 2015). We through the analysis of *cis*-acting elements in promoters of *PbPAL* family members, and found that most of the promoters of *PbPAL*s contain a variety of biological or abiotic stress-related elements (Table S5). Consequently, we hope to further study whether the hormones involved in these stress responses (SA, MeJA and ABA) could alter the expression of these genes (Fig. 6). After ABA treatments, the expression of *PbPAL1* was obviously induced, while the expression of *PbPAL2* was reversed, and the expression level was significantly inhibited. Interestingly, the expression of *PbPAL3* was induced at 1 and 3 h of treatment, but inhibited at 2 h, which the lowest expression level was found in 2 h of treatments (Fig. 6A).

In the MeJA-treated pear fruit, *PbPAL2* and *PbPAL3* showed the same trend, and were inhibited in 1 h and 3 h of treatments. After 2 h of treatments, they were significantly induced and the expression level reached peak. However, the expression level of *PbPAL1* showed an obvious opposite trend. The expression of *PbPAL1* was induced at 1 and 3 h of treatments, and the expression level reached peak at 3 h after treatments. After 2 h of treatments, the expression level was significantly inhibited (Fig. 6B).

The response patterns of *PbPAL*s to SA can be divided into two categories, including inhibiting gene expression and inducing gene expression. SA inhibited the expression of *PbPAL1* and *PbPAL2*, which was the lowest at 1 h. The other *PbPAL3* was induced by SA and peaked at 1 h with the prolongation of treatments time and the induction degree decreased (Fig. 6C).

### Determination of lignin content in transgenic *A. thaliana* of *PbPAL*s

**Figure 7:**
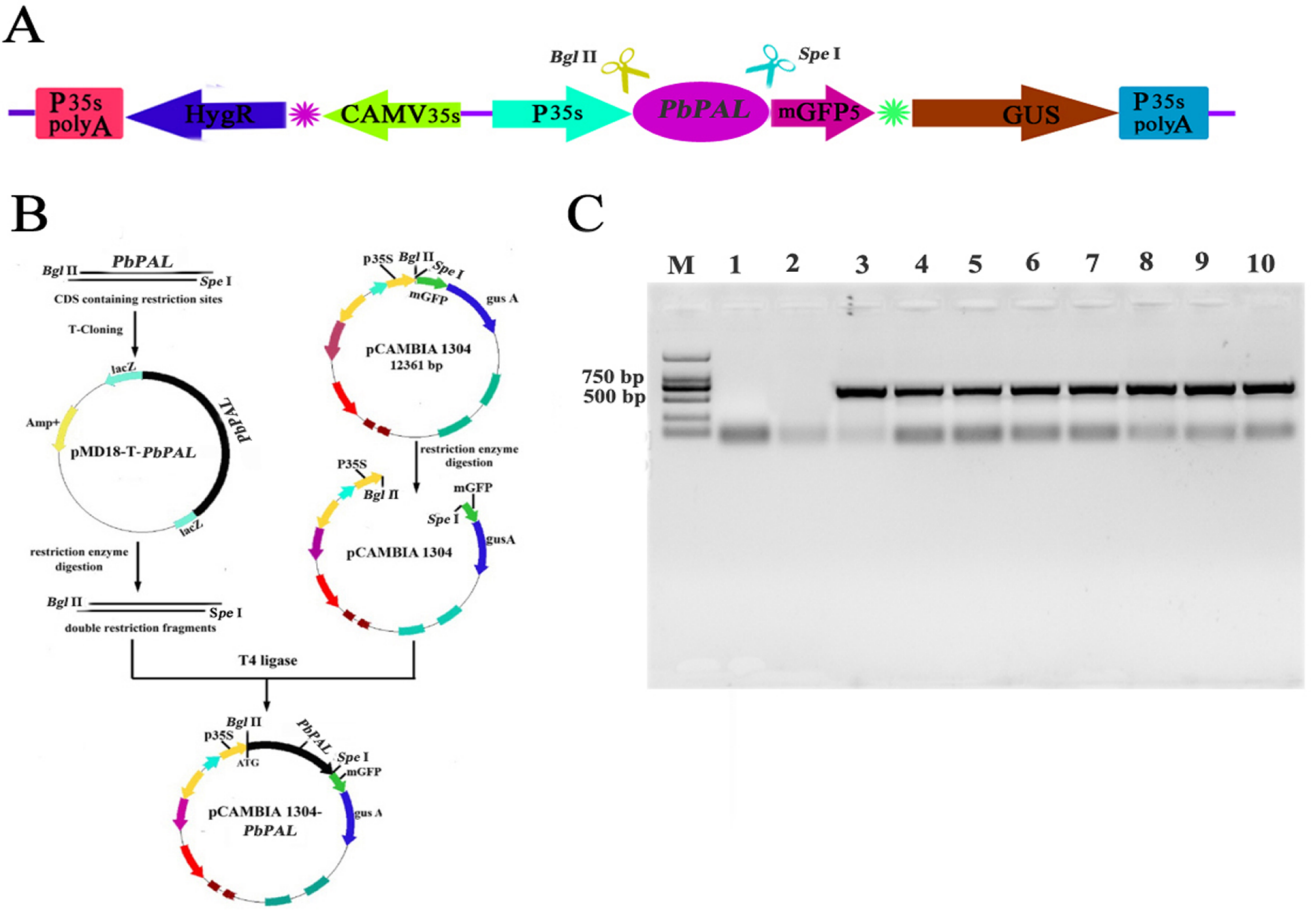
Vector construction and overexpression of *PbPAL1* and *PbPAL2* in Arabidopsis. (A) pCAMBIA1304-*PbPAL*. (B) *PbPAL*s were cloned and then inserted into the expression plasmid pCAMBIA1304-*PbPAL*. (C) The PCR analysis used specific primers to amplify the 700 bp internal fragment of *gfp*, M, DL2000 DNA Marker; 1-2, pure water; 3-4, pCAMBIA1304; 5-7, *PbPAL1* transgenic lines; 8-10, *PbPAL2* transgenic lines.

**Figure 8:**
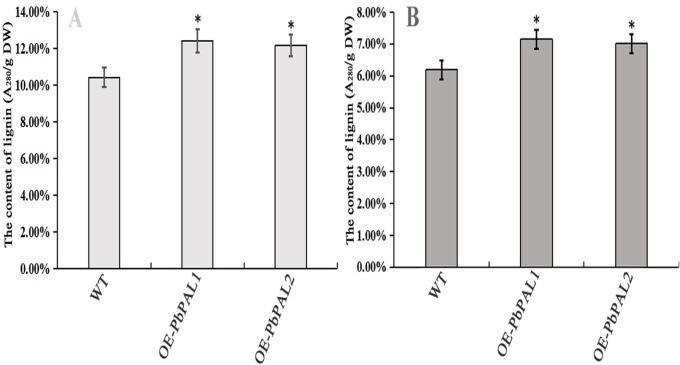
Determination of lignin content in *A. thaliana* stems and leaves. The lignin content of the transgenic plants was significantly different from that of wild-type plants (*P* < 0.05). (A) The lignin content of inflorescence stems. (B) The lignin content of leaves. WT, wild Arabidopsis; OE-*PbPAL1*, Overexpression of *PbPAL1* Arabidopsis; OE-*PbPAL2*, Overexpression of *PbPAL2* Arabidopsis. Error bar represents the standard error of three biological replicates.

To further determine the role of *PbPAL* genes in lignin synthesis, we obtained transgenic *A. thaliana* plants overexpressing these genes. Firstly, we constructed an eukaryotic expression vector (Fig. 7A). The DNA of the transgenic strain was amplified by GFP specific primers on pcambiA1304 vector (Fig. 7B). The successful cloning of the target fragment of approximately 700 bp indicated that the foreign gene has been successfully integrated into the *A. thaliana* genome (Fig. 7C). Subsequently, we successfully obtained three T_3_ generation transgenic lines of *PbPAL1* and *PbPAL2*. We determined the lignin level in *A. thaliana* inflorescence stems and leaves by acetyl bromide method (Fig. 8). The results showed that that the lignin content in inflorescence stems of transgenic plants of *PbPAL1* (12.42%) and *PbPAL2* (12.17%) was significantly higher than that of the wild-type plants (10.47%) (Fig. 8A). In addition, we determined that the lignin content in the leaves of transgenic *PbPAL1* (7.15%) and *PbPAL2* (7.01%) plants was also higher than that in wild-type *A. thaliana* (6.18%) (Fig. 8B). Our works demonstrated that both *PbPAL1* and *2* genes were involved in plant lignin synthesis.

### Lignin staining analysis

**Figure 9:**
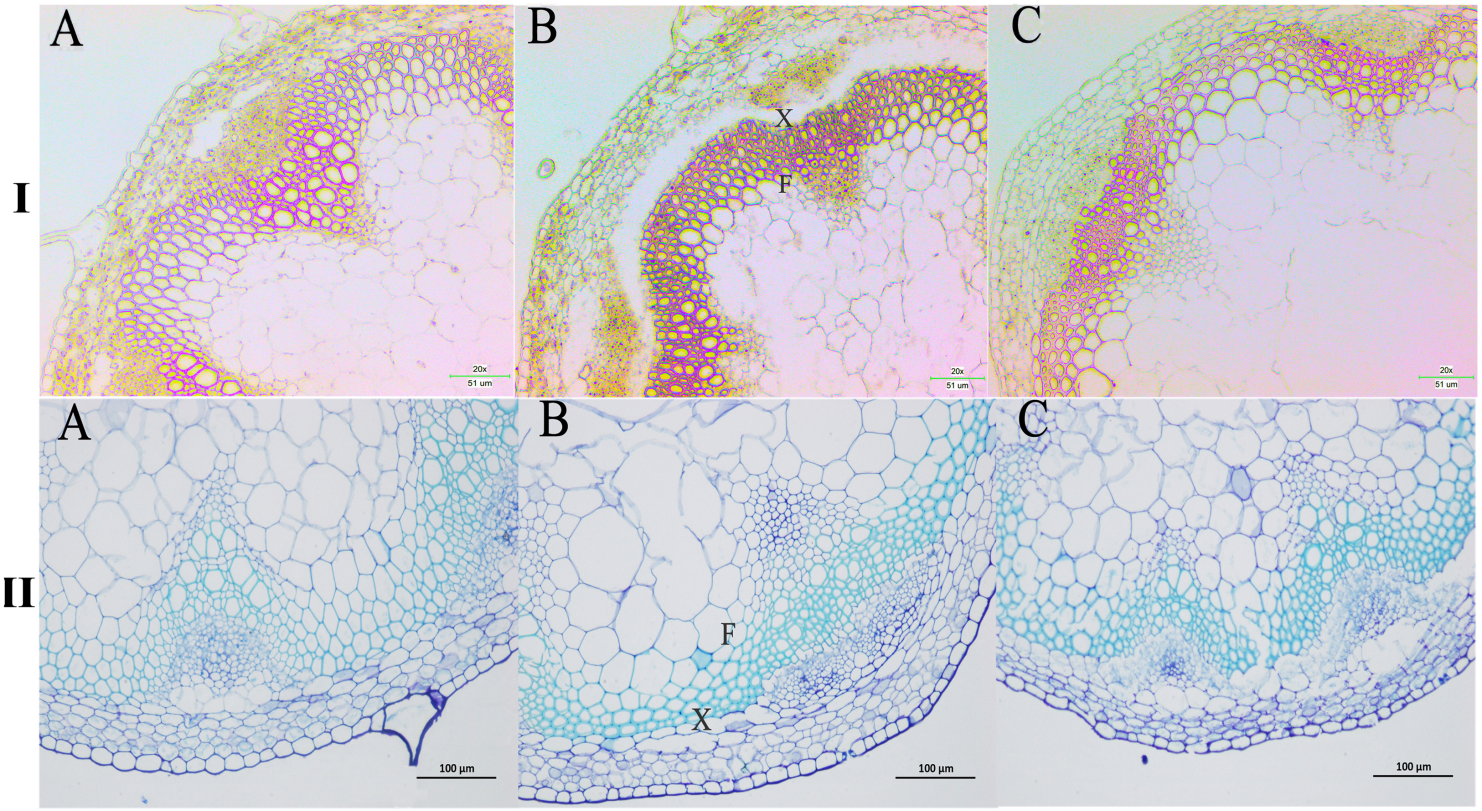
Wiesner and toluidine blue staining of cross-sections of *A. thaliana* inflorescence stems. All Arabidopsis plants were planted in the same environment; inflorescence stems were taken from *A. thaliana* plants and grown for 60 days. I refers Wiesner staining of cross-sections of *A. thaliana* inflorescence stems. II refers 1% toluidine blue staining of cross-sections of *A. thaliana* inflorescence stems. (A) WT plants. (B) *PbPAL1*-overexpressing transgenic plants. (C) *PbPAL2*-overexpressing transgenic plants. F, interfascicular fibre; X, xylem; bar = 51 µm and bar = 100 µm. B.

We next wanted to directly observe the distribution of lignin in inflorescence stems of transgenic *A. thaliana* plants. Thus, cross-sections of inflorescence stems of wild-type and transgenic plants were stained with phloroglucinol to identify possible changes in the content and/or distribution of lignified tissues. The Wiesner (phloroglucinol-HCl) staining results showed that the strongest staining of the xylem and intervascular fibers were observed in the stems of *PbPAL1* and *PbPAL2* transgenic *A. thaliana* than in wild-type plants (Fig. 9I). Furthermore, toluidine blue staining was used to examine the cell wall of a cross-sectional area of the *A. thaliana* pedicel (Fig. 9II). The cell wall thickness of *PbPAL1* and *PbPAL2* transgenic plants increased significantly. These two dyeing results showed that *PbPAL1* and *PbPAL2* can increase lignin synthesis. This is consistent with many previous studies, which *PAL* is related to the degree of lignification of plants (Chong et al., 2018).

## Discussion

The content and size of stone cells are the critical factors that affecting fruit quality and lignin plays a key role in the stone cells formation (Jin et al., 2013b; Li et al., 2017). Lignin deposits on the cell wall of pear fruits, making the secondary cell wall thicker (Cai et al., 2010; Tao et al., 2015). The present study found that there is a strong correlation between the formation of stone cells and lignin biosynthesis, which supports the view that lignin plays a vital function in stone cell biosynthesis (Jin et al., 2013b). Therefore, the study of lignin metabolism in pear fruits is of great significance for understanding the regulation of stone cell development. Lignin is produced by several metabolites of phenylpropanol (Rao et al., 2019; Wang et al., 2017). Phenylalanine ammonia lyase (PAL) is one of the key enzymes in lignin metabolism pathway (Starr et al., 2014). Therefore, screening and identifying *PbPAL*s related to lignin synthesis is important for understanding the regulation of lignin synthesis and stone cell development in pear.

In addition, PAL is also one of the branching enzymes linking primary and secondary metabolism (Ma et al., 2016). The first step in catalyzing cinnamic acid (a precursor) to form various phenylpropanol derivatives. In the present study, we identifified 16 *PAL*s from five Rosaceae species (Table 1). The number of *PAL*s in apple are nearly twice than that in pear, while the chromosome numbers of pear and apple were the same. Previous studies have revealed that genome-wide duplication of pear and apple offspring is based on whole-genome duplication (WGD) event learning in recent genome evolution processes (Xu et al., 2018). At the initial stage of evolution, the common ancestor of Rosaceae plants had nine chromosomes (Chong et al., 2018). Pear (*P. bretschneideri*) and apple (*M. domestica*) both experienced WGDs (Mya) and 30–45 Mya twice 130 million years ago, but only 17 chromosomes were found (Guo et al., 2013). This discovery indicated that the ancestors of the nine chromosomes of Rosaceae plants experienced doubling and breaking. After a long period of fusion, 17 chromosomes of pear and apple were finally formed. In this evolutionary process, the genome of a species may become very unstable, and it is ease to chromosome rearrangement, gene duplication and gene loss. In this process, the *PALs* in pear may be lost, which also explains why the number of *PAL*s in pear is much lower than that in apple.

Gene structure and conserved sequence construction may be intimately interrelated to the diversity of gene function (Cao et al., 2018). As anticipated, conserved domain analysis using these PAL protein sequences showed that genes of the same subfamily often had very similar genetic structures, suggesting that these genes might have similar functions (Fig. 1B). For example, *PmPAL2* and *PbPAL2* in Cluster III have the same genetic structure (two exons and one introns) and almost the same exon length. In addition, basing on the results of MEME analysis (Fig. 1C), we found that members of the same subfamily tend to have approximately the same conserved protein motif, but there are some differences in the motif composition among members of different subfamilies. We also found that some families contain specific conservative motifs, which means that these specific conservative motifs may be necessary for the specific functions of the subgroup, such as motifs 20 to Cluster I family.

Promoters regulate gene expression mainly at the transcriptional level and are coordinated by a variety of *cis*-acting elements and trans-acting factors (Soliman & Meyer, 2019). We discovered a great deal of hormone responsive *cis*-acting components in the upper reaches regulatory sequences of *PbPAL*s family members (Fig. 3 and Table S5). Especially, *PbPAL1* only contains abscisic acid (ABA)-responsive elements (ABREs) and *PbPAL2* only contains salicylic acid (SA)-responsive element. While abscisic acid (ABA)-responsive elements (ABREs), the methyl jasmonate (MeJA)-responsive element (CGTCA motif) and salicylic acid (SA)-responsive element (TCA element) were all found in *PbPAL3.* In addition, ethylene responsive elements (EREs) was only identified in *PbAL1* gene. These exogenous hormones are extensively participated in signaling pathways of mature aging or stress response (Betz, Mccollum & Mayer, 2001), which suggests that *PbPAL* family members might be involved in pear maturation and stress response.

In addition, we also found some *cis*-acting elements related to biological and abiotic stress in the upstream regulatory sequences of the *PbPAL*s, such as the TC-rich repeat elements (related to defence) and microtherm stress-related (LTR), and drought stress-related (MBS) elements (Table S5). These results suggested that *PbPAL* family members may play a role in response to various abiotic and biological stresses. Interestingly, we found that the promoter region of *PbPAL* family members of *PbPAL1* and *PbPAL3* have at least one AC element. AC element is a *cis*-acting element extensive consisting in the promoter region of lignin biosynthesis genes, such as *PAL*, *C4H* and *CAD* (Xu et al., 2014). It can activate lignin monomer synthesis gene by binding with MYB transcription factor (Cao et al., 2016). In addition, AC is in charge of the xylem-specific expression of lignin biosynthetic genes (Chong et al., 2018). Therefore, we founde that the AC elements in the upstream 2,000 bp putative promoter sequence of *PbPAL1* and *PbPAL3,* which hinted that they may be participated in the biosynthesis of pear lignin.

Gene expression patterns can provide important clues for exploring gene function (Budak & Zhang, 2017; Thomas et al., 2018). Previous researches have shown confirmed that the expression of the *PAL* genes were affected by exogenous hormone and salt in *C. sinensis* or drought stress in oil palm (Chong et al., 2018; Cao et al., 2016). To date, the role of *PAL* in pear fruit development is still unknown. Stone cell is one of the crucial factors affecting character of pear fruit and lignin is the essential constituent of stone cell (Yang et al., 2015). Previous studies have shown that content of stone cells in ‘Dangshan Su’ pear first increase and then decreases from 39 to 63 DAF, and the highest level was observed at 55 DAF (Cai et al., 2010; Chen et al., 2014). In this study, the qRT-PCR results shown that the *PbPAL1* and *PbPAL2* expression pattern showed a change tendency similar to that of the conent of lignin at different stages of pear fruit development. More importantly, we found that the expression of *PbPAL1* increased significantly at 55 DAF and showed a similar expression pattern to that of key genes participated in the regulation of lignin biosynthesis pathway (Xie et al., 2013). These results strongly hated that the *PbPAL1* and *PbPAL2* may regulate lignin synthesis in pear fruit. Besides, we found that the expression level of *PbPAL3* was low at early stage of pear fruit development, but higher in the late stages of fruit development. This is basically consistent with the expression level of *RiPAL2* in *Raspberry* (Ellis, 2001), which implies that *PbPAL3* plays an important role in the later stage of pear fruit development. These results suggested that the genetic diversity and functional differentiation of *PbPAL*s are necessary for plants to adapt to the environment.

Gene duplication events not only can promote the functional differentiation of *PAL* family during plant growth and development, but also *PAL* family under abiotic stress (Wu et al., 2017). For example, only *AtPAL1* and *AtPAL2* have functional specificity for nitrogen deficiency and low temperature in *A. thaliana* (Olsen et al., 2008). To understand the effect of abiotic stress on the expression level of *PbPAL*s. We analyzed *cis*-molecules in the upstream 2,000 bp putative promoter sequence of *PbPAL* family members and discovered that *PbPAL*s comprise a lot of elements responsive to ABA, SA and MeJA (Table S5), and studied the hormonal response pattern of *PbPAL*s. The results indicated that *PbPAL*s were induced or inhibited to varying degrees under several exogenous hormones treatments. MeJA can enhance disease resistance by stimulating plant defense mechanisms. Previous studies have reported that exogenous MeJA therapy enhances the induction of resistance, including the improvement of PAL activity in the phenylpropanol pathway (Wang et al., 2014a). In present study, the expression levels of three *PbPAL*s were all up-regulated after MeJA treatments. Therefore, the application of MeJA in pear fruit production can improve the disease resistance and content of phenylpropanoid compounds. The same gene expressed differently in different hormone treatments. Treating different genes with the same exogenous hormone results in similar or opposite expression trends of different genes. This indicated that the response pattern of *PbPAL* to hormones is very complex. We speculated that different *PbPAL*s play a role in different periods of time in adverse situation.

We have clearly known that some enzymes are involved in lignin synthesis. In some cases, appropriate genetic manipulations have altered the composition of lignin or reduced the content of lignin (Weng & Chapple, 2010). In many studies, *PAL*s have been found to be associated with lignification in plants. So far, very little has been reported about on lignin synthesis of pear *PAL* genes. Our results suggested that *PbPAL1* and *PbPAL2* may be involved in lignin biosynthesis in pears. Our hypothesis is further supported by the study of *PbPAL1* and *PbPAL2* in transgenic *A. thaliana*. The results showed that overexpression of *PbPAL1* and *PbPAL2* genes in *A. thaliana* increased the lignin content and cell wall thickness of plants. In future studies, we will transform the mutan *PbPAL*s into *A. thaliana* to further analyze its role in lignin synthesis.

## Conclusions

In present study, we screened and identified members of the *PAL* family from five Rosaceae genomes. In general, 16 *PAL*s were identified and three of them are from Chinese white pear. All *PAL*s are divided into three subfamilies on basis of phylogenetic analysis and structural characteristics of protein sequences. All *PAL*s were evenly distributed on 13 chromosomes. Gene duplication event analysis showed that segmental duplication played an important role in the expansion of *PAL* in Rosaceae species. Finally, qRT-PCR expression analysis showed that *PbPAL1* and *PbPAL2* might be involved in the formation of lignin and stone cells in pear fruits and transgenic experiments confirm the above conclusions.

PAL has many functions, our research focuses on the relationship between *PAL* and lignin and stone cells formation, which is a complete analysis of pear fruit. Heterologous expression of *PbPAL1* and *PbPAL2* in *A. thaliana* revealed that they are involved in lignin metabolism and cell wall growth. All in all, our observations can a provied basis understood of the five Rosaceae species’ *PAL* genes. Moreover, this research not only revealed the role of *PbPAL*s in lignin synthesis but also provided basic data needed to use molecular biology technonlogies to regulatelignin synthesis and stone cell development in pear.

##  Supplemental Information

10.7717/peerj.8064/supp-1 Figure S1 Sliding window analysis of *PAL* duplicated genes

10.7717/peerj.8064/supp-2 Table S1 Primer sequences used for qRT-PCR and vector construction

10.7717/peerj.8064/supp-3 Table S2 Sequences of specific primers for clone *PbPAL1* and *PbPAL2*.

10.7717/peerj.8064/supp-4 Table S3 Primer sequences contained artificial restriction enzyme sites for *Bgl* II and *Spe* I

10.7717/peerj.8064/supp-5 Table S4 * gfp* specific primers

10.7717/peerj.8064/supp-6 Table S5 Numbers of *cis*-elements in promoter region of *PbPAL*s

10.7717/peerj.8064/supp-7 Table S6 Geme name gene ID in this study

10.7717/peerj.8064/supp-8 Data S1 Raw data PAL-Different tissue (Bud-Stem-Leaf-Flower); PAL-Different tissue (Root); PAL-Fruit development at different stages (15, 39, 47, 55, 63, 79, 102, 145 DAF); PAL-Hormone treatment; The content of stone cells and lignin.

## Additional Information and Declarations

### DNA Deposition

The following information was supplied regarding the deposition of DNA sequences: The Cluster A sequences are available at: Phytozome (https://phytozome.jgi.doe.gov/pz/portal.html): - *Malus domestica*: MDP0000261492, MDP0000388769, MDP0000139075, MDP0000191304; - *Prunus persica*: Ppa002099m, Ppa002878m, Ppa002328m - *Prunus mume*: Pm018524, Pm030127 - *Fragaria vesca*: Fv09753, Fv23261 Rosaceae (https://www.rosaceae.org): - *Pyrus communis*: P018639.1, P043094.1, P027603.1, P008617.1, P019735.1, P026924.1. GIGADB (http://gigadb.org/dataset/100083): - *Pyrus bretschneideri*: Pbr016460.1, Pbr008363.1, Pbr008387.1 Phytozome (http://www.phytozome.net/poplar): - *Populus trichocarpa*: 010G0224100.1, 010G0224200. NCBI: XP_006381441.1, XP_002322884.2, NP_001047482, AAK76593, CAB64229, AAS18574, AAF02809. The Cluster B sequences are available at: NCBI: ADB97626, XP_003580144, XP_004976241, ACF86361, NP_001151482, XP_003575400, XP_003575238, XP_003575240, XP_003575403, XP_003575404, XP_003575365, NP_001055608, ACN27043, XP_008645952, ACN62413, A2X7F7, NP_001047484. The Cluster C sequences are available at: NCBI: DAA36917, XP_008668474, XP004976238, XP_004958719, EAZ31356, NP_001053324, AAO72666, S06475, CAA34226, NP_001047481, ADE08261, XP_003575396, XP_004976240, ACL53545, P11544.2, WP_012408693.1, WP_007245619.1, XP_004973667, XP_008678828, Q8GMG0.1, WP_011339422.1, WP_016502005.1, ACN24505, XP_011048538.1.

### Data Availability

The following information was supplied regarding data availability: Raw data are available in the Supplemental Files.
