# Supplementary figures and images for: Comparative genomic analysis of the PAL genes in five Rosaceae species and functional identification of Chinese white pear
Source: PeerJ. 2019 Dec 2;7:e8064. doi: 10.7717/peerj.8064 (PMC6894436; doi:10.7717/peerj.8064)

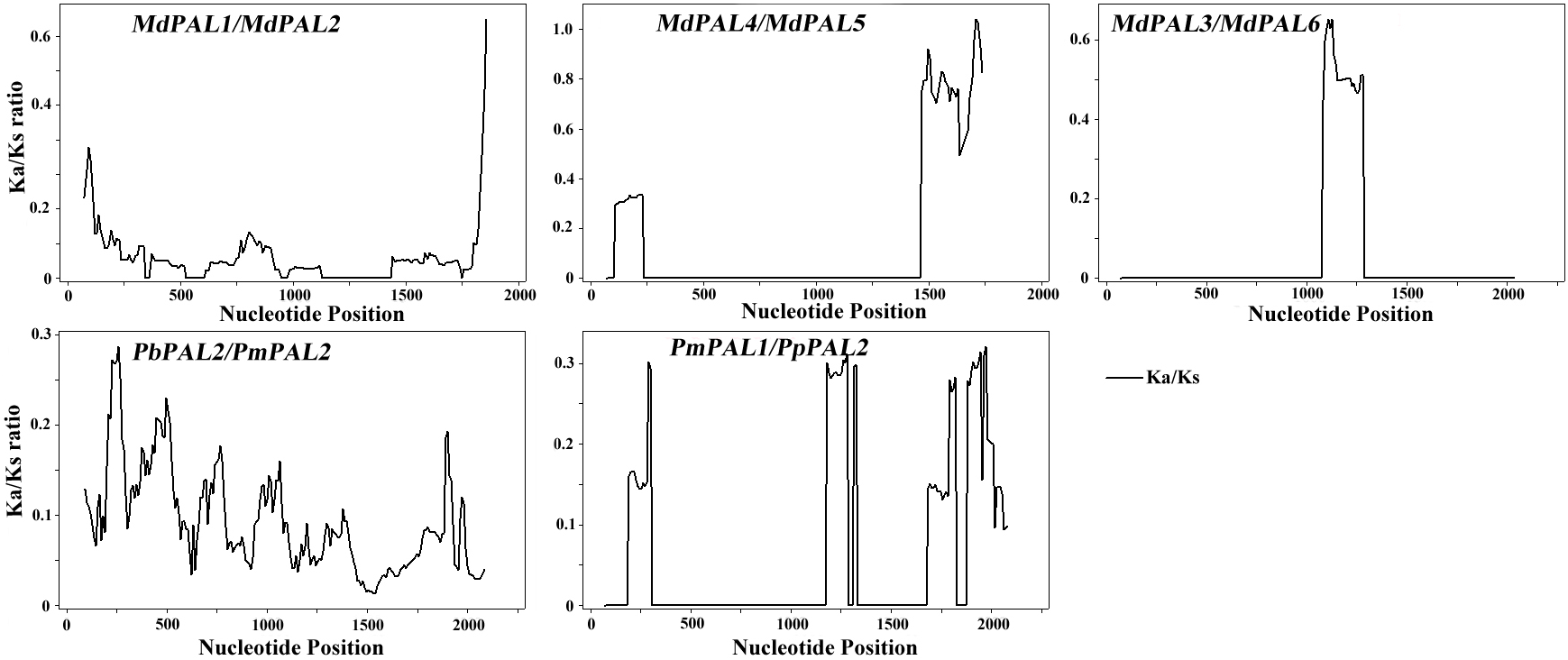

Supplement: Figure S1 [file peerj-07-8064-s001.png]
