# Supplementary material for: Comparative genomic analysis of the PAL genes in five Rosaceae species and functional identification of Chinese white pear
Source: PeerJ. 2019 Dec 2;7:e8064. doi: 10.7717/peerj.8064 (PMC6894436; doi:10.7717/peerj.8064)
Supplement: Table S1 [file peerj-07-8064-s002.doc]

**Table S1 Primer sequences used for qRT-PCR.**

| Gene name | Upstream primer （5′→3′） | Downstream primers （5′→3′） |
| --- | --- | --- |
| *PbPAL1* | GAAGTGCTACAGAATCAG | GAATCTTATGCCAGAGTAG |
| *PbPAL2* | ATCTCCATCCATCAAGGT | TACAAGGACAGAATGTTCAC |
| *PbPAL3* | AAGATTGGAGCTTTCGAGGA | TCTGTTCCAAGCTCTTCCCT |
| Tublin | AGAACAAGAACTCGTCCTAC | GAACTGCTCGCTCACTCTCC |
