# Supplementary material for: Comparative genomic analysis of the PAL genes in five Rosaceae species and functional identification of Chinese white pear
Source: PeerJ. 2019 Dec 2;7:e8064. doi: 10.7717/peerj.8064 (PMC6894436; doi:10.7717/peerj.8064)
Supplement: Table S2 [file peerj-07-8064-s003.doc]

**Table S2 Sequences of specific primers for clone *PbPAL1* and *PbPAL.***

| Gene name | 5′→3′ |
| --- | --- |
| *PbPAL1*-F | ATGGAGGCGGAAACCATCACC |
| *PbPAL1*-R  I-F | CTAACAGATAGGAAGAGGTGCGC |
| *PbPAL2* -F | ATGGCTTCTGAGCTAGCTTCAG |
| *PbPAL2*-R  I-F | TTAACATATTGGAAGGGGACTG |
