# Supplementary material for: Comparative genomic analysis of the PAL genes in five Rosaceae species and functional identification of Chinese white pear
Source: PeerJ. 2019 Dec 2;7:e8064. doi: 10.7717/peerj.8064 (PMC6894436; doi:10.7717/peerj.8064)
Supplement: Table S3 [file peerj-07-8064-s004.doc]

Table S3 Primer sequences contained artificial restriction enzyme sites for *Bgl* II and *Spe* I

| Gene name | 5′→3′ |
| --- | --- |
| *PbPAL1* *Bgl* II-F | GA**AGTCT**ATGGAGGCGGAAACCATCAC |
| *PbPAL1 Spe* I-R  I-F | GC**GTCGAC**CTAACAGATAGGAAGAGGTG |
| *PbPAL2* *Bgl* II-F | GA**AGTCT**ATGGCTTCTGAGCTAGCTTC |
| *PbPAL2 Spe* I-R  I-F | GC**GTCGAC**TTAACATATTGGAAGGGGACT |
