# Supplementary material for: Comparative genomic analysis of the PAL genes in five Rosaceae species and functional identification of Chinese white pear
Source: PeerJ. 2019 Dec 2;7:e8064. doi: 10.7717/peerj.8064 (PMC6894436; doi:10.7717/peerj.8064)
Supplement: Table S4 [file peerj-07-8064-s005.doc]

**Table S4 *gfp* specific primers.**

| Primer name | Primer sequence (5*'*~3*'*) |
| --- | --- |
| *gfp*-F | GGAGAAGAACTTTTCACTGG |
| *gfp*-R | GTAATCCCAGCAGCTGTTAC |
