# Supplementary material for: Comparative genomic analysis of the PAL genes in five Rosaceae species and functional identification of Chinese white pear
Source: PeerJ. 2019 Dec 2;7:e8064. doi: 10.7717/peerj.8064 (PMC6894436; doi:10.7717/peerj.8064)
Supplement: Table S5 [file peerj-07-8064-s006.doc]

**Table S5 Numbers of *cis*-elements in promoter region of *PbPAL*s.**

| Element | MBS | MRE | AC | LTR | HSE | TC-rich repeat | ABRE | ERE | CGTCA motif | TCA-element | Total |
| --- | --- | --- | --- | --- | --- | --- | --- | --- | --- | --- | --- |
| Function | Drought stress | Light response | MYB  bindin | Low-temperature stress | Heat stress | Defense and stress | ABA response | Ethylene response | MeJA response | SA response |
| *PbPAL1* | 1 | 1 | 2 | 1 |  |  | 5 | 1 |  |  | 11 |
| *PbPAL2* | 3 | 1 |  |  |  |  |  |  |  | 2 | 6 |
| *PbPAL3* |  |  | 3 | 1 |  |  | 1 |  | 2 | 2 | 9 |
| Total | 4 | 2 | 5 | 2 |  |  | 6 | 1 | 2 | 4 | 26 |
