# Supplementary material for: Comparative genomic analysis of the PAL genes in five Rosaceae species and functional identification of Chinese white pear
Source: PeerJ. 2019 Dec 2;7:e8064. doi: 10.7717/peerj.8064 (PMC6894436; doi:10.7717/peerj.8064)
Supplement: Table S6 [file peerj-07-8064-s007.doc]

Table S6 Geme name gene ID in this study

| **Gene name** | **Gene ID** |
| --- | --- |
| ZmPAL1 | NP_001241797 |
| ZmPAL2 | ACL53545 |
| ZmPAL3 | DAA36917 |
| ZmPAL4 | XP_008668474 |
| ZmPAL5 | XP_008678828 |
| ZmPTAL6 | NP_001105334 |
| ZmPAL7 | ACN27043 |
| ZmPAL8 | ACF86361 |
| ZmPAL9 | NP_001151482 |
| ZmPAL10 | XP_008645952 |
| SiPAL1 | XP_004953153 |
| SiPAL2 | XP_004976240 |
| SiPAL3 | XP_004973667 |
| SiPAL4 | XP004976238 |
| SiPAL5 | XP_004958719 |
| SiPAL6 | XP_004976241 |
| BoPTAL1 | ADE08261 |
| BoPAL2 | ACN24505 |
| BoPAL3 | ACN62413 |
| BoPAL4 | ADB97626 |
| BoPAL8 | XP_003580144 |
| PcPAL1 | P018639.1 |
| PcPAL2 | P008617.1 |
| PcPAL3 | P043094.1 |
| PcPAL4 | P027603.1 |
| PcPAL5 | P026924.1 |
| PcPAL6 | P019735.1 |
| PtPAL1 | XP_006381441.1 |
| PtPAL2 | XP_011048538.1 |
| PtPAL3 | XP_002322884.2 |
| PtPAL4 | Potri.010G0224100.1 |
| PtPAL5 | Potri.010G0224200.1 |
| OsPAL1 | NP_001047481 |
| OsPAL2 | NP_001053324 |
| OsPAL3 | S06475 |
| OsPAL4 | CAA34226 |
| OsPAL5 | AAO72666 |
| OsPAL6 | EAZ31356 |
| OsPAL7 | A2X7F7 |
| OsPAL8 | NP_001047484 |
| OsPAL9 | NP_001047482 |
| OsPAL10 | NP_001055608 |
| AthPAL1 | AAK76593 |
| AthPAL2 | CAB64229 |
| AthPAL3 | AAS18574 |
| AthPAL4 | AAF02809 |
| RspTAL | WP_011339422.1 |
| PspHAL | WP_016502005.1 |
| SgTAM | Q8GMG0.1 |
| PsHAL | WP_007245619.1 |
| RtTAL | P11544.2 |
| NpPAL | WP_012408693.1 |
| BdPTAL1 | XP_003575396 |
| BdPAL2 | XP_003575400 |
| BdPAL3 | XP_003575403 |
| BdPAL4 | XP_003575404 |
| BdPAL5 | XP_003575365 |
| BdPAL6 | XP_003575238 |
| BdPAL7 | XP_003575240 |
| PbPAL1 | Pbr008363.1 |
| PbPAL2 | Pbr008387.1 |
| PbPAL3 | Pbr016460.1 |
| PpPAL1 | Ppa002328m |
| PpPAL2 | Ppa002099m |
| PpPAL3 | Ppa002878m |
| PmPAL1 | Pm030127 |
| PmPAL2 | Pm018524 |
| FvPAL1 | Fv23261 |
| FvPAL2 | Fv09753 |
| MdPAL1 | MDP0000668828 |
| MdPAL2 | MDP0000787168 |
| MdPAL3 | MDP0000261492 |
| MdPAL4 | MDP0000388769 |
| MdPAL5 | MDP0000139075 |
| MdPAL6 | MDP0000191304 |
